# Large-area and adaptable electrospun silicon-based thermoelectric nanomaterials with high energy conversion efficiencies

**DOI:** 10.1038/s41467-018-07208-8

**Published:** 2018-11-12

**Authors:** Alex Morata, Mercè Pacios, Gerard Gadea, Cristina Flox, Doris Cadavid, Andreu Cabot, Albert Tarancón

**Affiliations:** 1 IREC, Catalonia Institute for Energy Research, Department of Advanced Materials for Energy, Jardins de les Dones de Negre 1, Planta 2, Sant Adrià del Besós, 08930 Barcelona, Spain; 2 Universidad Nacional de Colombia, Departamento de Física, Ciudad Universitaria, Bogotá, 111321 Colombia; 3 ICREA, Passeig Lluís Companys 23, 08010 Barcelona, Spain

## Abstract

Large amounts of waste heat generated in our fossil-fuel based economy can be converted into useful electric power by using thermoelectric generators. However, the low-efficiency, scarcity, high-cost and poor production scalability of conventional thermoelectric materials are hindering their mass deployment. Nanoengineering has proven to be an excellent approach for enhancing thermoelectric properties of abundant and cheap materials such as silicon. Nevertheless, the implementation of these nanostructures is still a major challenge especially for covering the large areas required for massive waste heat recovery. Here we present a family of nano-enabled materials in the form of large-area paper-like fabrics made of nanotubes as a cost-effective and scalable solution for thermoelectric generation. A case study of a fabric of p-type silicon nanotubes was developed showing a five-fold improvement of the thermoelectric figure of merit. Outstanding power densities above 100 W/m^2^ at 700 °C are therefore demonstrated opening a market for waste heat recovery.

## Abstract (web summary)

To realize waste heat recovery solutions based on thermoelectricity, high-performance materials with device and manufacturing compatibility are required. Here, the authors demonstrate large-area paper-like nanostructured fabrics consisting of aligned nanotubes with high thermoelectric performance.

## Introduction

From the human body and combustion engines to volcanoes and hot industry exhausts, waste heat sources are present everywhere. Turning this residual heat into an exploitable form of energy such as electricity is a major challenge for scientists and engineers. In this sense, using the thermoelectric effect to generate electricity from thermal energy sources has been explored for many years. Thermoelectric generators present exceptional advantages like simplicity, size and power scalability, adaptability to different temperature ranges and a long-time stability facilitated by the absence of mobile parts. Nevertheless, waste heat recovery strategies based on thermoelectricity are not massively implemented yet due to the high cost and scarcity of the raw materials currently used and the low efficiency and poor scalability of the resulting devices.

The goodness of a thermoelectric material is commonly measured with the dimensionless figure of merit ZT:

1 $${\mathrm{ZT}} = \frac{{\sigma {S}^2}}{\kappa }{T},$$

where *σ* is the electrical conductivity, *k* is the thermal conductivity, and *S* is the Seebeck coefficient. Electrical and thermal conductivities tend to be coupled in nature, in such a way that it is not trivial to find materials that conduct electrons efficiently but remain thermal insulators. Indeed, few families of materials with a ZT maximum around 1 dominated the thermoelectric field from until the 90s, namely, Bi_2_Te_3_-based materials for applications around room temperature (up to 450 °C), PbTe-based materials for use in an intermediate temperature range (up to 850 °C) and SiGe for use at the highest temperatures (up to 1350 °C). It was not until the 90s and 2000s that new strategies to achieve quality thermoelectric materials were explored, exploiting novel properties appearing at the nanoscale^1–7^. Theoretical calculations and experimental evidences proved that the performance of some materials can be significantly enhanced when nanostructuring them. Among other, it is remarkable the proof that silicon, a nontoxic and highly abundant element, can show excellent thermoelectric performance when in the form of nanowires and nanotubes^8,9^. Unfortunately, the implementation of these new families of nanomaterials into real devices remains a challenge due to some factors that go beyond the figure of merit^10,11^. Major issues that have to be solved prior implementing these outstanding properties in real products include the small-area coverage, low adaptability, and the difficult cost-effective scalability of current manufacturing processes^12^.

In this work, we present a family of highly performing nanostructured thermoelectric metamaterials produced by an industrially scalable manufacturing method. This procedure allows the fabrication of large-area fabrics in the form of easy-to-handle paper-like sheets adaptable to surfaces of arbitrary shapes.

The resulting thermoelectric material shows a significant improvement of the figure of merit ZT (ZT = 0.34 at 550 °C), five times lower than bulk doped silicon. More interestingly, the fabrics have been tested in simple harvesting modules reaching power densities of 110 W m^−2^ at 700 °C,

## Results

### Microstructure and fabrication procedure

**Fig. 1:**
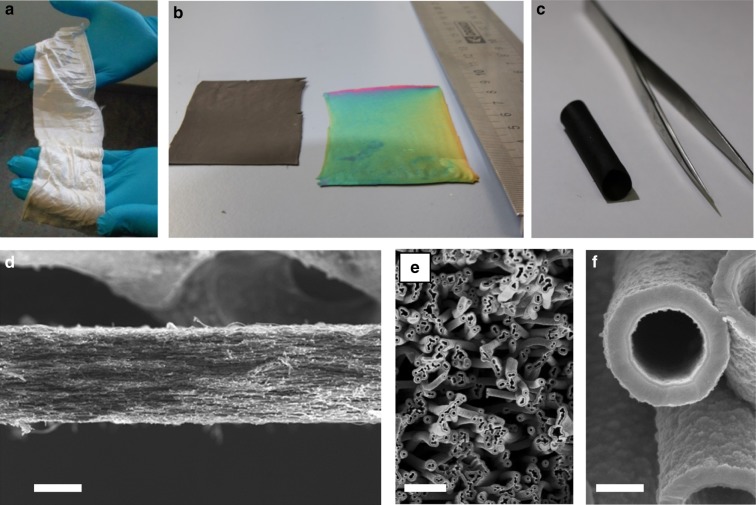
Illustrative images of the morphology of the poly-silicon (pSi) nanotube fabrics at each fabrication step. As-electrospun fabrics of polymer (**a**), the carbon nanofiber-based fabrics after carbonization (**b**, **c**) and the silicon nanotube-based fabrics after the whole fabrication process (**b**). Scanning electron microscope cross-sectional images of thermoelectric fabric at different scales with scale bars corresponding to **d** 50 µm, **e** 5 µm, **f** 500 nm

Figure 1 shows a general overview at different scales of the presented material which is made of fabrics of partially aligned polycrystalline nanotubes arranged in a three-dimensional porous microstructure. This architecture ensures low thermal diffusivity across the fabrics by enhancing phonon scattering at the nanotube walls and strongly restricting the thermal convection across the array of micropores. This multi-scale scattering approach leads to an overall reduction of the thermal conductivity without the need of complex vacuum encapsulation. Moreover, the interconnected nature of the three-dimensional nanotube network prevents the presence of interfaces typically constituting charge trapping centers that lead to drastic reductions of the electrical conductivity of porous materials^13^.

**Fig. 2:**
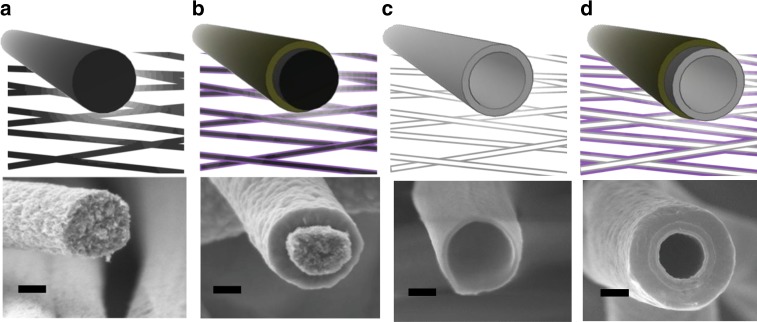
Detailed scheme of the fabrication steps and images of the corresponding representative scanning electron microscope . Carbon fiber (**a**), poly-silicon layer coating the carbon fiber (**b**), silicon oxide substrate remaining after the annealing of the carbon core (**c**), poly-silicon layer surrounding the silicon oxide substrate (**d**). Scale bars are 200 nm long

In this work, as a case study, we use this approach to develop large-area and cost-effective boron-doped poly-silicon fabrics, following the methodology defined hereafter. The fabrication method consists in using carbon-based fabrics prepared by electrospinning (Fig. 2a) as a sacrificial support for the deposition of thin silicon layers by chemical vapor deposition (CVD). These thin shells of silicon form nanotubes after removal of the carbon template (Fig. 2b–d). The sacrificial carbon templates have been fabricated from electrospun polyacrylonitrile (PAN) fabrics annealed at 750 °C in reducing atmosphere. Sheets of 5 × 10 cm made of aligned carbon nanofibers of 600 nm in diameter (Fig. 2b) were coated with doped silicon in a large-area low-pressure CVD. A poly-silicon layer of 30 nm in thickness is grown on top of the as-generated fibers. An annealing process in air is carried out in order to eliminate the sacrificial carbon^14^, naturally generating Si oxide nanotubes (Fig. 2c). This silicon oxide is used as the final support for subsequent thermoelectric poly-silicon layers (Fig. 2d). The thickness of these silicon layers can be straightforwardly controlled by the CVD deposition time, being 65 nm in the current case. Details on the fabrication process conditions can be found in the Methods section. The morphology of the metamaterial is presented in detail in electron microscopy images included in Supplementary Figure 1 and Supplementary Note 1, in which a crystalline Si shell is clearly observed on top of an amorphous SiO_*x*_ substrate. X-ray diffraction of the fabrics (shown in Supplementary Figure 2) demonstrates the polycrystalline nature of the thermoelectric Si layer. In terms of mechanical and thermal stability, the silicon-based fabrics present a consistency similar to paper, being easily adaptable to multiple geometries (Fig. 1b, c) and an excellent stability at temperatures as high as 700 °C (Supplementary Figures 3 and 4; Supplementary Note 2). These features allow their potential application in high-temperature scenarios requiring small- and even large-area waste heat recovery such as industrial environments presenting hot surfaces at 400–500 °C. In this direction, the proof-of-concept fabrics produced in the current work have been fabricated involving well established mass manufacturing techniques such as electrospinning and CVD^15,16^.

### Thermoelectric characterization

**Fig. 3:**
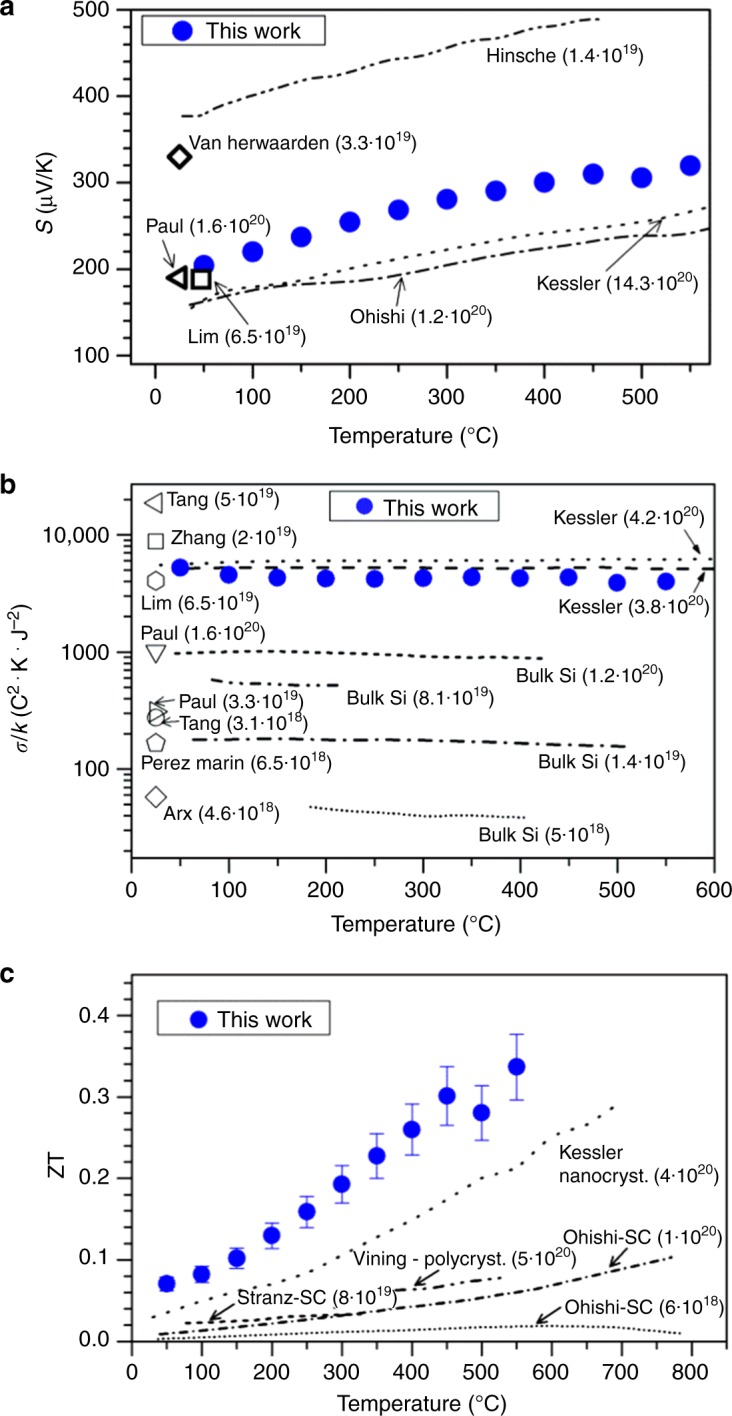
Seebeck coefficient, *σ*/*k* and figure of merit (ZT) of the fibers of nanotubes. Seebeck coefficients *S* as a function of the temperature *T* (**a**). The blue circles represent the fabric of this work, with nanotube wall thickness of 65 ± 5 nm. Reported values at room temperature pSi thin films with different doping levels from Paul et al.^17^ and Van Herwaarden et al.^18^ are included. Lines represent the values expected for bulk/nanobulk pSi, from Hinsche et al.^19^, Ohishi et al.^20^, and Kessler et al.^22^. Values in brackets indicate the boron doping concentration of the respective materials. **b** Geometry-independent figure *σ*/*k* as a function of temperature for the same pSi NT fabric (black squares). Values obtained from literature have been included in the graph for comparison: holey silicon nanostructures^21^^,^^30^, etched silicon^22^, thin films^17,]^^31,32^, pressed nanocristalline silicon^23^. **c** Figure of merit of the samples with different wall thickness as a function of temperature. Dash lines from bulk crystalline Si^20^^,^^28^ and polycristalline Si^28^. Dot line represents the values obtained from the work of Kessler et al.^23^. The main source of error reflected in the bars in the plot of ZT comes from the uncertainty in the  measurement of the effective section, required for   determining both *σ* and *κ*. It has been derived from the standard error of SEM cross sections obtained at different regions of the measured sample

In order to fully characterize the intrinsic thermoelectric properties of the nano-enabled silicon-based fabrics, the Seebeck coefficient (*S*) and the thermal (*κ*) and electrical (*σ*) conductivities were measured. Figure 3a shows *S* as a function of the temperature in the range from room temperature to 550 °C. For comparison, the same graph includes values reported in the literature for similar doping levels^17–23^. The values obtained for our fabrics are in the expected order of magnitude for highly boron-doped poly-silicon samples. Being *S* almost unaffected by the porosity and sample microstructure, this parameter gives us an indication of the doping levels achieved in our synthesis process, which is in the range of 10^20^ cm^3^. Supplementary Figure 5 presents a more precise comparison with the literature using room temperature values and plotting boron-doped silicon at different doping concentrations.

Independent values of *σ* and *κ* can be found in the Supplementary Figure 6 and Supplementary Note 3 and is compared with different literature values^17,21,22,24–26^. A remarkable decrease observed for both *κ* and *σ* is due to the trivial effect of a high porosity and tortuosity. This is typically observed in materials in which the conduction pathway that forces electrons and phonons to move through a propagation route not crossing the pores (see sketch in Supplementary Figure 6a)^27–29^.

The ratio *σ*/*κ* is independent of the porosity and tortuosity of the sample and can be used for determining the real gain associated to a particular nanostructure by comparing the benefits of the reduction of the thermal conductivity with the drawback of the loss of electrical conductivity. The ratio *σ*/*κ* is presented in Fig. 3b for the nano-Si-based fabrics as a function of the temperature. The values of *σ*/*κ* measured in this work are c.a. 4000 C^2^K J^−2^ in the whole range of temperature. These values are similar to the ones reported by Lim et al.^21^. for highly doped poly-Si thin films (6.5 × 10^19^ cm^−3^) or by Kessler et al.^22^. for nanocrystalline bulk Si (4 × 10^20^ cm^−3^) while three-to-four times higher than highly doped bulk crystalline silicon. This significant improvement compared to the bulk counterpart is clearly observed in Supplementary Figure 7 and Supplementary Note 3 where the factor *σ*/*κ* as a function of boron doping concentration is specifically represented. The origin of this improvement in *σ*/*κ* compared to the bulk is the reduction in a factor ~3300 of the thermal conductivity, which approaches 50 Wm K^−1^ for the bulk and 0.015 W mK^−1^ in the Si fabrics at RT^20,30^, while *σ* presents a three-fold decrease, ca. 80,000 S m^−1^ for the bulk compared to ca. 80 S m^−1^ for the fabrics at RT.

Combining previously presented Seebeck coefficient and *σ*/*κ* values, the figure of merit (ZT) corresponding to this material is represented in Fig. 3c. The values range from 0.07 at 25 °C to 0.34 at the maximum temperature measured in this work, i.e. *T* = 550 °C. The same graph includes reported values for bulk and different Si nanostructures for direct comparison^20,22,31,32^. A five-fold increase of the ZT value is obtained for the silicon nanotube fabrics referred to the silicon bulk demonstrating the benefits of the nanostructuring approach presented here. Moreover, a relevant 50% of improvement in ZT is obtained with this methodology compared to values reported by Kessler et al. for nanocrystalline silicon. The ZT values reported here are among the best obtained for a silicon-based macroscopic material, with the additional advantage that our silicon-based fabrics are shape-adaptable and produced by large-scale fabrication techniques. The high ZT values obtained here for large-area and adaptable fabrics demonstrate the potential of extending the range of application of nanostructured silicon from very specific niche applications such as micro-thermoelectric generators to a wide spectrum of large-scale scenarios including industrial waste heat recovery^33,34^.

### Thermoelectric power generation

**Fig. 4:**
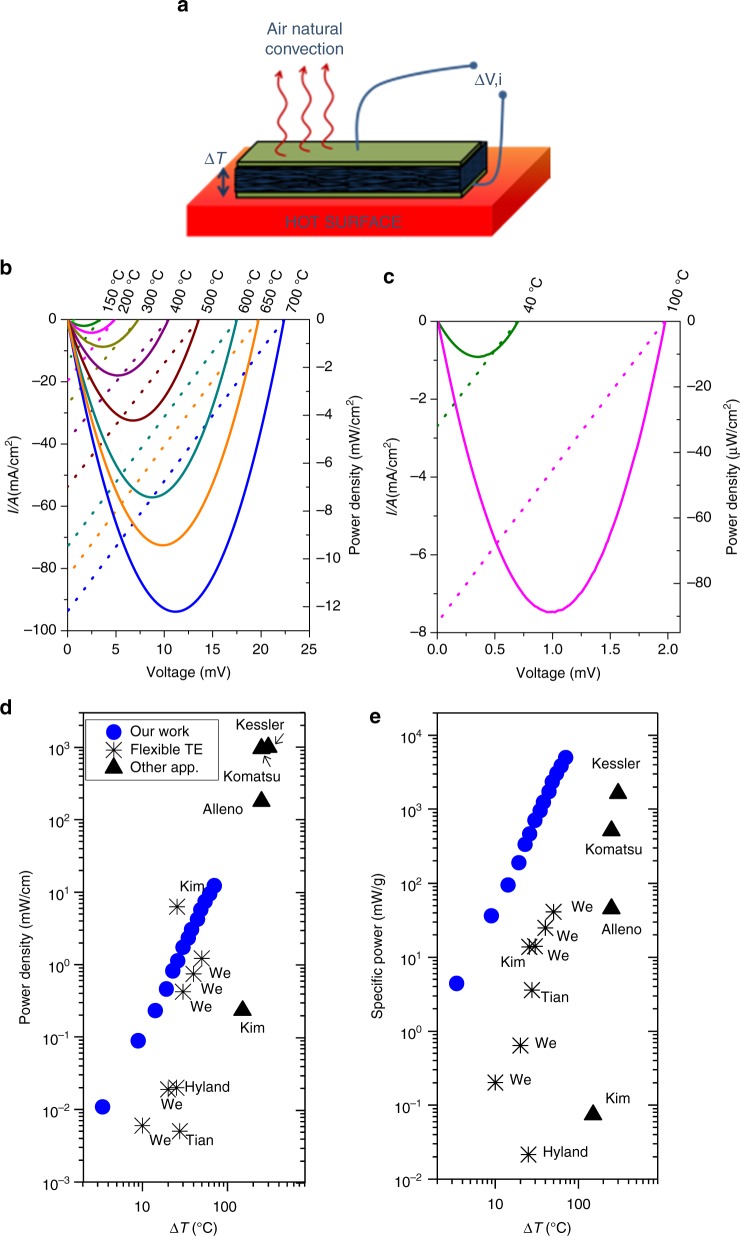
Thermoelectric generation of the material at different temperatures. Sketch representing the test configuration (**a**). Voltage–current (dash lines) and power (full lines) curves from a sheet of silicon nanotubes fibers in contact with a surface in the high (**b**) and low (**c**) temperature range. Maximum power per unit area (**d**) and weight (**e**) obtained. Some other well documented works are presented as references for different temperature ranges and applications. Flexible thermoelectrics^34–38^, other higher scale applications ^22,33,38^, and commercial products like *Komatsu*^40^

In order to directly prove the potential of this metamaterial in realistic energy harvesting operating conditions, nano-Si based fabrics were implemented as a component in a simple thermoelectric generation module. This test configuration consisted in a sheet of the material (p-doped Si nanofibers) with Mo contacts in both layers put in contact with a hot surface, set at temperatures in the range of 40 to 700 °C (see Methods section for information on this experiment). Figure 4b, c shows the voltage–current and power–current response for these modules based on a 70-µm-thick fabric of silicon fibers contacted with Mo pads on both sides. In the sake of clarity, high- and low-temperature ranges have been split in Fig. 4b, c, respectively. In these measurements, the open circuit voltage (OCV) indicates the gradient of potential generated by the module while the maximum power represents the highest electrical output generated by the system due to its internal resistance. Obviously, higher values of OCV and power density were naturally achieved when increasing the temperature of the hot surface. For temperatures of 700 °C, typically occurring in industrial waste heat recovery scenarios and solar concentration applications^35,36^, remarkable OCV values of 22 mV (equivalent to a gradient of temperature Δ*T* = 70 °C generated across a distance as small as 70 µm) and elevated power densities of *P* = 11 mW cm^−2^ (i.e. 110 W m^−2^) were achieved. In the low-temperature range of 40 °C, characteristic of the human body and with application in wearable and implantable technologies, an open circuit voltage of 0.7 mV (Δ*T* of 3.5 °C) was reached, providing a maximum power density of 10.8 µW cm^−2^. According to these output values, the nano-Si fabrics show an excellent performance in the whole range of temperature when compared to the state-of-the-art thermoelectric power generation devices reported so far^37–44^. Such outstanding results in an extended temperature range are related to the extremely low thermal conductivity of the material, which allows a relatively high thermal gradient to be sustained across a very thin layer. This large thermal gradient translates into a considerable voltage difference, while the reduced thickness of the layer does not impose a too elevated electrical resistance for practical applications. To contextualize the obtained results, the power density as a function of the temperature difference achieved was represented in Fig. 4d, e and compared with reference energy conversion devices. The fabric based on silicon nanotubes here presented is one of the first examples of competitive silicon-based thermoelectric generator since most of the works previously reported in the literature (and used for comparison in Fig. 4d) are based on expensive and critical raw materials such as Bi_2_Te_2.7_Se_0.3_^36^, Bi_2_Te_3_^37,40^, Bi_2_Te_3_-Sb_2_Te_3_^34^, and Eu_8_Ga_16_Ge_30_–Sr_0.5_Fe_4_NiSb_12_^33^. Furthermore, thanks to the high porosity of the sample, the provided specific power is remarkably high (Fig. 4e), which provides an advantage for applications, like aerospace power generation, in which the weight of the final system is of major importance.

## Discussion

In this work, a paradigm for developing highly efficient thermoelectric materials in the form of large area and adaptable fabrics made of partially aligned nanotubes has been presented. This approach has been successfully proved for the particular case of silicon, which is a champion material with key features such as abundance, low toxicity, biocompatibility, and well established fabrication routes. The resulting nano-enabled thermoelectric material showed a remarkable five-fold improvement of the figure of merit ZT (ZT = 0.34 at 550 °C) compared to the bulk counterpart. More interestingly, the fabrics have been tested in simple modules reaching thermoelectric power density generation of 110 W m^−2^ in harvesting mode at industrially relevant temperatures of 700 °C, which is comparable to other reference power generation techniques. Moreover, the universality of the method, which can be implemented using different materials, and the high tunability of the nanosized features (i.e. the wall thickness of the nanotubes that control the ZT enhancement by drastically decreasing the thermal conduction) anticipate further advances through the use and optimization of other materials and nanostructures. This room for improvement and the cost-effective and scalable techniques employed point to future impact of this paradigm in the deployment of thermoelectric materials in commercial applications.

## Methods

### Fabrication process

The steps for fabricating poly-silicon (pSi) nanotube fabrics are illustrated in Fig. 2. We depart from electrospun carbon nanofibers that are used as a first sacrificial template. These are covered with Si by a first low-pressure chemical vapor deposition (LP-CVD) process, generating fibers with a carbon core and a pSi shell. A calcination is performed in an external oven to remove the carbon. This process produces a partial oxidation of the pSi. A second CVD process within the CVD system is conducted in order to deposit the active pSi layer.

The base substrate employed herein is a fabric of carbon nanofibers obtained from annealing of polyacrylonitrile (PAN) nanofibers produced by electrospinning. These were synthesized by means of an electrospinning equipment (Nanotechnology Solutions, Yflow®), where fibers are extruded from a negatively charged polymer-containing droplet and oriented to a positively metallic collector by the high voltage applied between them. A rotating collector in combination with motion along the rotating axis allowed to endow the nanofibers a high degree of uniformity in areas up to 2000 cm^2^. The PAN fibers were carbonized by a conventional two-step process comprising: (i) an oxidative stabilization annealing of 250 °C in air during 7 h, to remove hydrogen by means of a cyclization process of PAN; (ii) a carbonization/partial graphitization process in argon at 750 °C during 1 h which removes nitrogen leaving behind carbon fibers shown at Fig. 2a. Details on the process can be found elsewhere^45^.

Si and SiGe growth was carried out by means of Vapor-Solid CVD technique using a commercial FirstNano Easitube 3000 low-pressure CVD system. SiH_4_ gaseous precursor is used for the deposition of Si, both for the substrate nanotubes and the active layer. A dopant precursor (B_2_H_6_ in this proof of concept) is always introduced, aiming the highly doped films required for the thermoelectric application.

The carbon fibers were loaded into the CVD reactor for a VS deposition process of a pSi thin film layer on top. The conditions of the CVD-VS deposition are shown in Table 1. After deposition the carbon fibers presented a thin pSi coating of 30 nm. These fibers are composed of pSi and carbon. In order to remove the thermoelectrically not performant carbon a calcination was carried at 900 °C during 3 h. The combustion of the carbon takes place, leaving behind a hollow silicon oxide structure, as shown in Fig. 2c. This thin and hollow SiO2/pSi structure is loaded in the reactor again, repeating the CVD-VS thin film growth which leads to the finally functioning thermoelectric fiber shown at Fig. 2d. This last functional pSi shell was again grown at the conditions show in Table 1 to form the final functional material. Four hundred nanometer Mo layers are sputtered at both sides of the sheets as electrical contacts (Ac450 Alliance Concepts).

**Table 1:** CVD conditions for VS pSi thin film deposition

Parameter	SiO_2_ substrate	Si active layer
Temperature (°C)	630	630
Pressure (Torr)	5	1
Time (min)	30	300
SiH_4_-H_2_ flow (sccm)	20	10
200 ppm B_2_H_6_ in H_2_ flow (sccm)	50	50

### Structural characterization

Scanning electron microscope (SEM) (ZEISS Auriga) with a In-Lens detector was used to characterize the morphology of the samples, using voltages of 1.5 keV. X-ray diffraction (XRD) was used for the determination of the crystal structure of the samples, also allowing to confirm the Si–Ge ratio in the alloy. The XRD system was a Bruker AXS D8 ADVANCE with Cu *K*_α1_ radiation (*λ* = 1.5406 Å).

### Thermoelectric characterization

Electric properties: The cross-plane electrical conductivity and the measurement of the power curves were derived from *I*–*V* curves obtained in a four wires configuration using a Keithley 2400 source meter. A sketch of the measurement configuration is shown in Supplementary Figure 5a The fibers were cut into squares of 6 mm^2^ and located on top of a Linkam gas-tight chamber equipped with a heating stage that allows precisely imposing temperatures up to 700 °C. Five percent hydrogen in argon was introduced in order to protect the Mo contacts. Fifteen minutes stabilization time was imposed prior to every measurement to ensure thermal equilibrium. The same measurement setup was used to determine the power output achieved with the material in a harvesting mode configuration (see sketch in Fig. 4a). The temperature difference appeared naturally by the insulating nature of the nano-sheets in contact with the heater and no extra forced convection was applied. The temperature difference was determined from the OCV and the Seebeck coefficient. Δ*T* at temperatures higher than 550 °C was approximated using *S* at 550 °C. The downward trend of *S* at increased temperatures might produce Δ*T* underestimation. Seebeck coefficient of pSi NT fabrics was measured with Linseis LSR 3 System using a static DC method. A sample is vertically positioned between two clamps that behave as electrodes. The entire arrangement is located in a furnace that sets the temperature from room temperature to 550 °C, while a heater positioned in one of the clamps imposes a small temperature gradient (c.a. 4 K). Two thermocouples contact the sample laterally. In order to give enough mechanical strength to the samples to overcome the stresses from the clamps and the thermocouple spring constants, the fabric is attached to glass supportive pieces and are contacted on both ends with silver paste and aluminum caps.

Thermal properties: The thermal diffusivity (*α*) of the pSi NT fabrics was measured by laser flash analysis (LFA) using a Linseis LFA 1000 apparatus in a vacuum atmosphere. Thermal conductivity (*κ*) was obtained by the application of the defining formula *κ* = *αρC*_*p*_, being *C*_*p*_ the specific heat of the material, obtained from literature and considering the relative amounts of Si and SiO_2_^46,47^. For the determination of the density (*ρ*), the sample mass was measured by using a high precision microbalance from a Perkin Elmer TGA4000 system. The sample thickness was obtained by means of a precision caliper (Mitutoyo Digimatic) and corroborated with SEM cross-sectional images. The area was measured with an optical microscope provided with Dino Capture 2.0 analysis software that delivers the exact area of the samples after calibration.

### Durability tests

Durability tests at high temperature under air were performed in a ceramic test station (Probostat^TM^) inserted into a tubular furnace. In this case, silver contacts were painted at both sides of the sheet and connected with gold cables. The resistance of the sample submitted to 600, 700, and 800 °C was tracked during 24 h.

## Electronic supplementary material

Supplementary Information

## Data Availability

The datasets generated during and/or analyzed during the current study are available in the Figshare repository [10.6084/m9.figshare.6903275].
