# Supplementary material for: Large-area and adaptable electrospun silicon-based thermoelectric nanomaterials with high energy conversion efficiencies
Source: Nat Commun. 2018 Nov 12;9:4759. doi: 10.1038/s41467-018-07208-8 (PMC6232086; doi:10.1038/s41467-018-07208-8)
Supplement: Supplementary file 1 — Supplementary Information [file 41467_2018_7208_MOESM1_ESM.pdf]

Supplementary Information

Large-area and adaptable electrospun Silicon-based thermoelectric nanomaterials with high energy conversion efficiencies

Morata et al.

Supplementary Figures

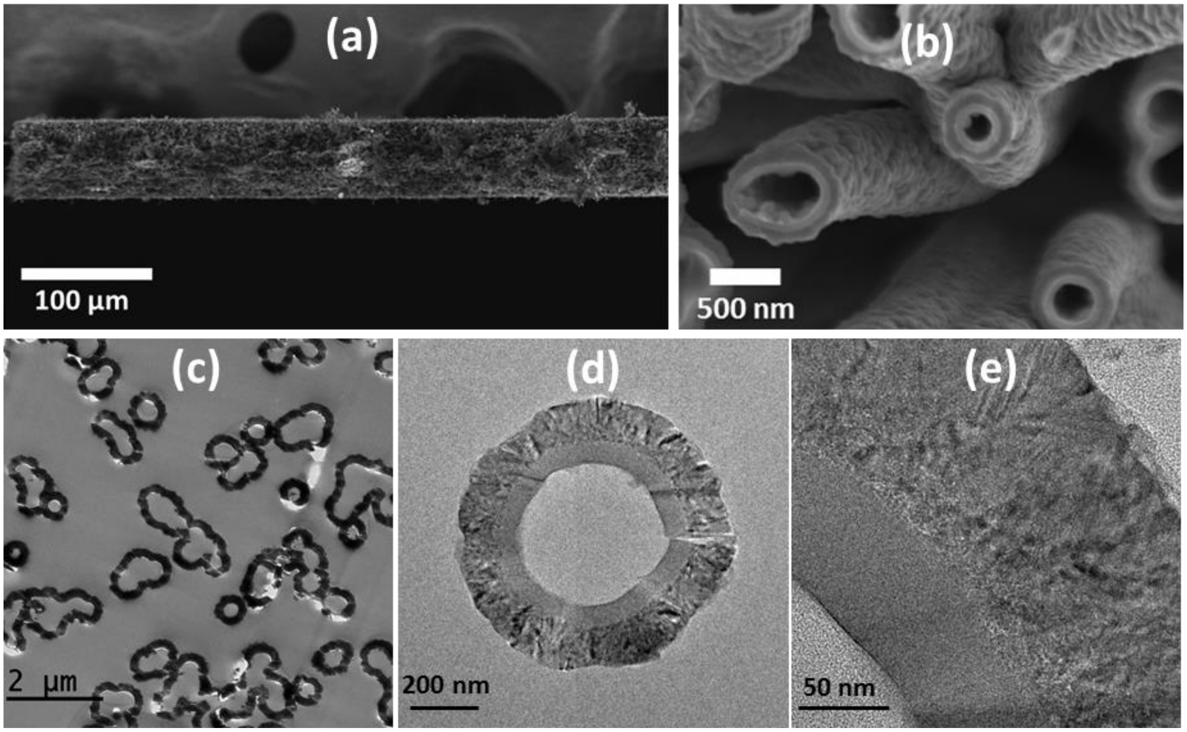

Supplementary Figure 1. Morphology of the samples. SEM images of the sheet's cross section (a, b). TEM images from slides of the sample previously embedded in resin at different magnifications (c-e).

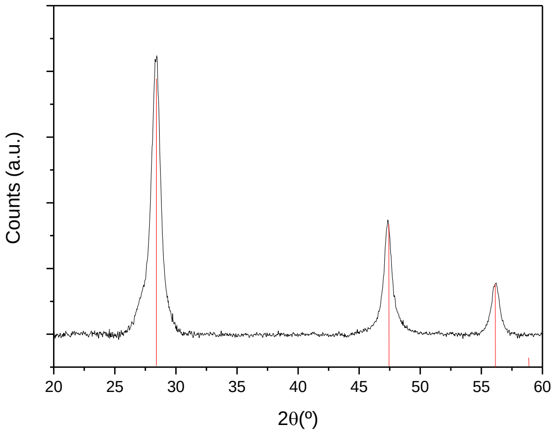

Supplementary Figure 2: XRD of the silicon material conforming the fibers. The results demonstrate the poly-crystalline nature of the nanotubes. Simple analysis by means of the Scherrer equation provides an average crystallite size of 18±3 nm.

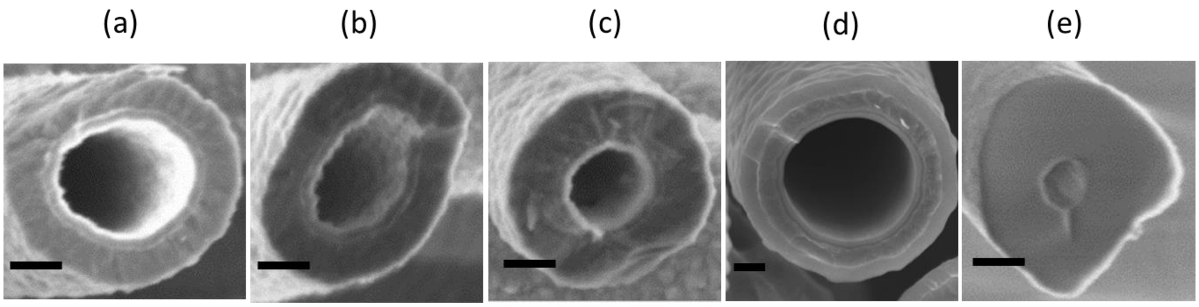

Supplementary Figure 3: SEM images of Si nanotubes at room temperature (a) and after overcoming an annealing process at 500 °C (b), 700 °C (c), 800 °C (d) and 1000 °C (e). The annealing time is 24h in all the cases except for the sample exposed to 800°C, in which it is 48h. Scale bars are 200 nm long.

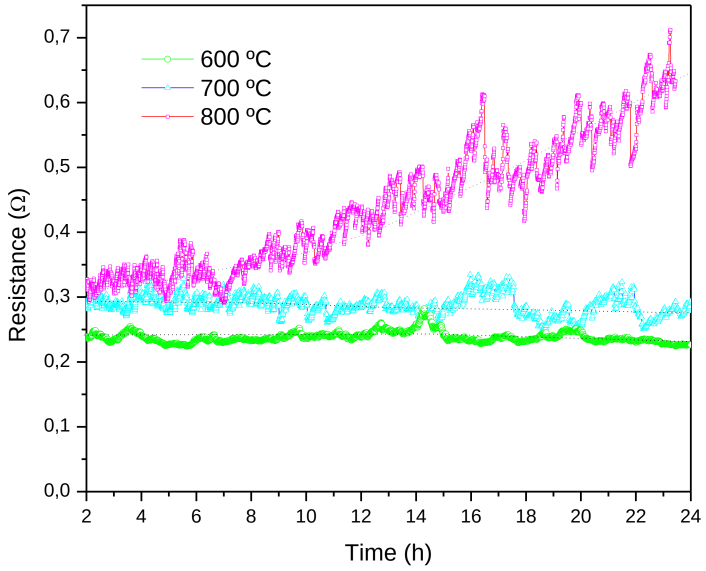

Supplementary Figure 4: Evolution of resistance of a sheet of Si nanotubes at different imposed temperatures. Black dotted lines are guides for the eyes.

.

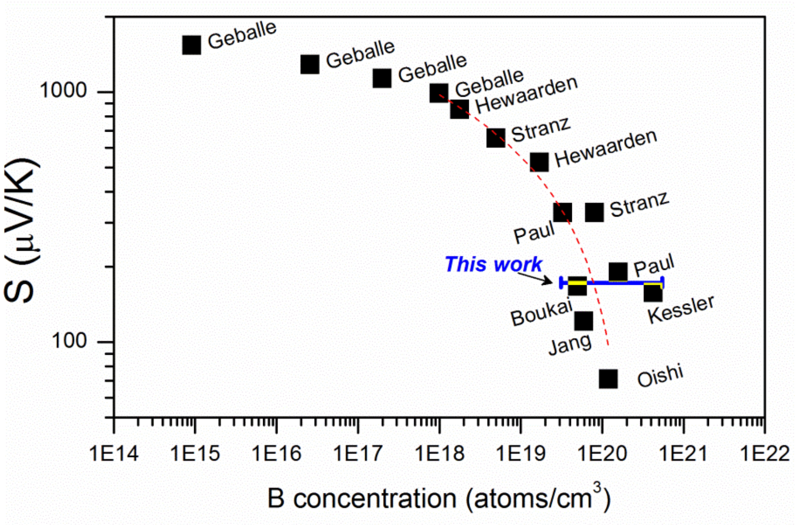

Supplementary Figure 5: Seebeck coefficient at room temperature as a function of boron concentration measured in different works in literature. The blue line indicates the value of the Seebeck coefficient at room temperature obtained in this work. As can be observed, despite the variability of the bibliographic data, we can approximate to have a doping concentration ranging between $5\cdot10^{19}$ and $5\cdot10^{20}$ cm^{-3}.References from Geballe et al.[1], Hewaarden[2], Stranz et al.[3], Paul et al.[4], Kessler et al.[5], Boukai et al.[6], Jang et al.[7], Ohishi et al.[8].

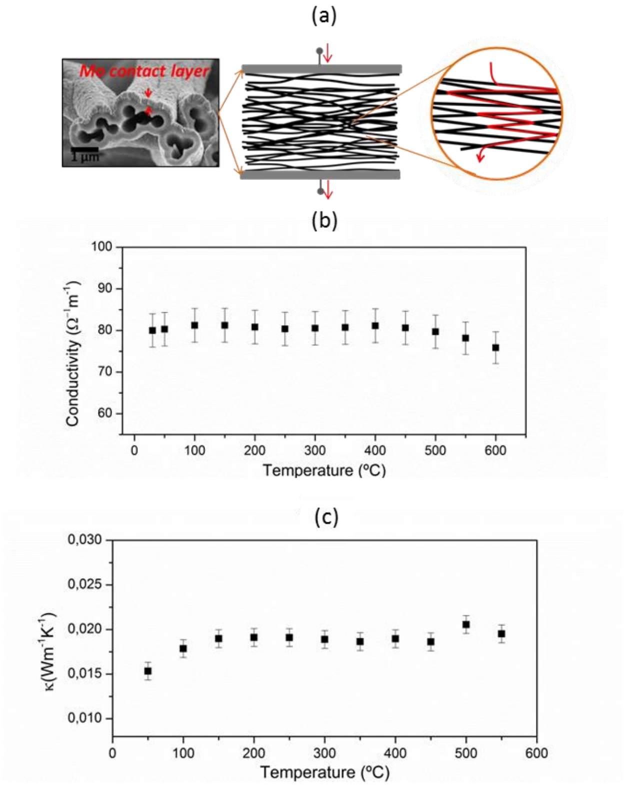

Supplementary Figure 6: Sketch of the measurement configuration. Detail of the Mo contact layer and sketch of the expected tortuous conduction paths (a). Electrical conductivity of the material sheet measured in cross section at different temperatures using both side Mo contact layers and platinum contacts (b). Thermal conductivity of the material sheet measured in cross section by the Laser Flash method (c).

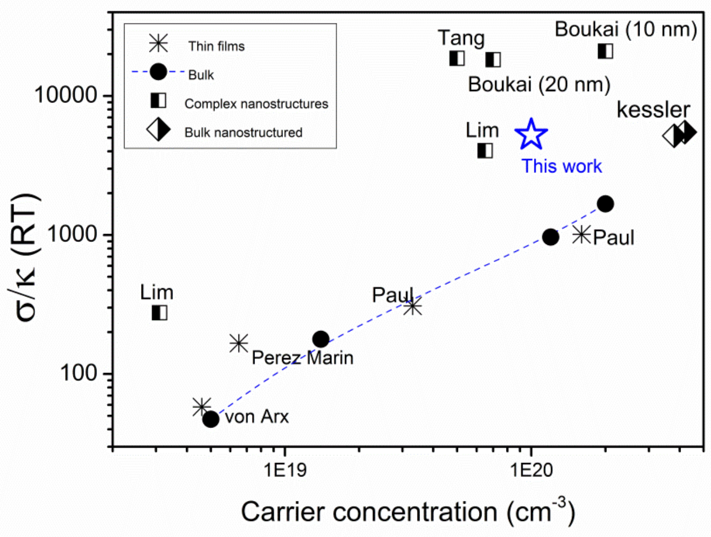

Supplementary Figure 7: Comparison of the room temperature σ/κ values obtained in this work with different references from literature: Tang et al.[9], von Martin et al.[10], Kessler et al.[5], Perez Marin et al.[11], Lim et al.[12], Paul et al.[4] and Hochbaum et al.[13]

Supplementary Notes:

Supplementary Note 1: Supplementary Figure 1 shows the morphology of the samples in cross section at different levels. A macroscopic view of the sample (Supplementary Fig. 1a) shows a relatively homogeneous thickness all along the sheet, presenting values of 70 ± 5 µm. A higher magnification SEM picture of a transversal surface (Supplementary Fig. 1b) reveals a clear direction of alignment of the nanotubes, often attaching each other forming bundles. This frequent attachment can be important as it might ease the electrical connection between the top and bottom surfaces of the sheets. The amorphous core and the crystalline shell are clearly distinguishable. In order to improve the sample characterization it is often convenient to observe a 2D section of the sheets. TEM images of a slice of the Si nanotube fibres obtained in a direction perpendicular to the sheet are presented in Supplementary Fig. 1c-e.

Both SEM and TEM pictures show relatively wide distribution on the tube diameter, corresponding to the sacrificial carbon nanofibers, presenting an average diameter is around 300 nm. In contrast, the thickness of the silicon oxide substrate and the deposited active silicon layer are better defined, showing values of 50 ± 5 nm and 70 ± 15 nm, respectively. A certain difference of thickness is however observed between the nanotubes close to the surface of the sheet and the ones situated in a deeper region. The porosity of the sample can be estimated from this image, leading to a value of 83 ± 2 % which corresponds to a density value of 0.36 ± 0.07 g/cm^3. This result is compatible with the one measured by means of a high precision microbalance, using an optical microscope and a high precision caliper to determine the dimensions (0.35 ± 0.03 g/cm^3).

Supplementary Note 2: In order to have an indication of the stability of the material, the fibers were submitted to 24h annealing processes at different temperatures. As can be seen in the SEM images Supplementary Fig. 3, the tubular structure of the samples is preserved in all the considered cases. However, at 800 °C a SiO_2 shell is clearly visible and at 1000°C a full oxidation of the fibers takes place. No evident changes are observed at 700°C or below. These observations are in concordance with the evolution of the electrical resistance of the samples subjected to high temperature under air (see Supplementary Fig. 4). While the resistance is maintained constant on samples subjected to 600 and 700 °C, there is a clear progressive enhancement when they are exposed at 800 °C.

Supplementary Note 3: The electrical and thermal conductivity measured in the sample are presented in Supplementary Fig. 6. The values obtained in both cases are far below the expected for poly-silicon, as is expected from porous samples with highly tortuous conduction paths. The actual thermal and electrical conductivity of the active material deeply depends on the complex geometry of the sample in the microscale, making impossible to directly compare these values with literature. However, as both magnitudes are expected to be equally affected by the geometry at this scale, the factor σ/k can serve for this purpose. The most complete comparison with other works can be carried out at room temperature, where profuse bibliography exists for boron-doped silicon prepared in different ways. Supplementary Fig. 7 shows different approaches for Si fabrication: thin films, nanostructured bulk and other more complex structures including nanowires. The values arising from the here presented silicon nanotube fibers are clearly above the expected for bulk silicon, approaching the most outstanding improvements obtained from complex nanostructuring techniques.

Supplementary References

[1] Geballe, T., Hull, G., Seebeck Effect in Silicon, *Phys. Rev*., **98**, 940–994 (1955)
[2] Van Herwaarden. A.W., The Seebeck Effect in Silicon ICs., Sensors and Actuators, **6**, 245-254, (1984)
[3] Stranz, A., Waag, A., Peiner, E., High-temperature performance of stacked silicon nanowires for thermoelectric power generation, *J. Electron. Mater*., **42**, 2233–2238 (2013).
[4] Paul, O., Von Martin, A., Baltes, H., Process-dependent thermophysical properties of CMOS IC thin films, *Proceedings of the International Solid-State Sensors and Actuators Conference*, 1, 178–181 (1995)
[5] KesslerV., et al., Fabrication of High-Temperature-Stable Thermoelectric Generator Modules Based on Nanocrystalline Silicon *J. Electron. Mater*., **43**, 1389–1396 (2014)
[6] Boukai, A. I., et al., Silicon nanowires as efficient thermoelectric materials, Nature, **451**, 168–171(2008).
[7] Jang, M., et al., The Characteristics of Seebeck Coefficient in Silicon Nanowires Manufactured by CMOS Compatible Process Nanoscale *Res. Lett.*, **5**, 1654–1657 (2010).
[8] Ohishi, Y. et al, Thermoelectric properties of heavily boron- and phosphorus-doped silicon, *Jpn. J. Appl. Phys.*, **54**,71301 (2015).
[9] Tang, J., et al., Holey Silicon as an Efficient Thermoelectric Material, *Nano Letters*, **10**, 4279–4283 (2010).
[10] Martin, von A., Paul, O., Baltes, H., Process-dependent thin-film thermal conductivities for thermal CMOS MEMS, *Journal of Microelectromechanical Systems*, **9**, 136–145 (2000)
[11] Perez-Marín, A.P., et al., Micropower thermoelectric generator from thin Si membranes, *Nano Energy*, **4**, 73–80 (2014)
[12] Lim, J.et al., Simultaneous Thermoelectric Property Measurement and Incoherent Phonon Transport in Holey Silicon, *ACS Nano,* **10**, 124-132 (2015)
[13] A.I. Hochbaum, A.I., et al., Enhanced thermoelectric performance of rough silicon nanowires., *Nature*, **451**, 163-167 (2008)
